# Autologous Bone Marrow Stem Cells in the Treatment of Chronic Liver Disease

**DOI:** 10.1155/2012/307165

**Published:** 2011-11-03

**Authors:** Madhava Pai, Duncan Spalding, Feng Xi, Nagy Habib

**Affiliations:** ^1^Department of HPB Surgery, Hammersmith Hospital, Imperial College, Hammersmith Campus, London, UK; ^2^Faculty of Medicine, Imperial College London, Hammersmith Campus, Du Cane Road, London W12 0NN, UK

## Abstract

Chronic liver disease (CLD) is increasing worldwide yet there has been no major advance in effective therapies for almost five decades. There is mounting evidence that adult haematopoietic stem cells (HSC) are capable of differentiating into many types of tissue, including skeletal and cardiac muscle, neuronal cells, pneumocytes and hepatocytes. These recent advances in regenerative medicine have brought hope for patients with liver cirrhosis awaiting transplantation. New findings in adult stem cell biology are transforming our understanding of tissue repair raising hopes of successful regenerative hepatology. Although all clinical trials to date have shown some improvement in liver function and CD34^+^ cells have been used safely for BM transplantation for over 20 years, only randomised controlled clinical trials will be able to fully assess the potential clinical benefit of adult stem cell therapy for patients with CLD. This article focuses on the potential of bone marrow stem cells (BMSCs) in the management of CLD and the unresolved issues regarding their role. We also outline the different mechanisms by which stem cells may impact on liver disease.

## 1. Introduction

The liver has a remarkable capacity to regenerate in response to injury; however, in severe cases its regenerative capacity may prove to be insufficient, and the liver injury may progress to end-stage liver disease (ESLD) and subsequent liver failure. Up to two million people suffer from chronic liver disease in the UK, many of whom remain unaware of their illness [1, 2]. Chronic liver disease is the fifth leading cause of death in the UK after cancer, cardiovascular disease, stroke, and respiratory disease [2]. Alcoholic liver disease (ALD), one of the major medical complications of alcohol abuse, is the commonest cause of ESLD in Europe and North America and is one of the most controversial indications for transplantation. Alcohol abuse accounts for 80% of all liver cirrhosis cases seen in district general hospitals in the United Kingdom [3] and for a substantial and increasing proportion of all liver transplants performed. Chronic liver disease (CLD) due to alcohol abuse continues to rise [4]. In 2005, 4,160 people died in England and Wales from alcoholic liver disease, an increase of 37% since 1999 [1]. Alcoholic liver cirrhosis (ALC) has an unfavourable prognosis, with a mortality of 49% and 90% after 1 and 15 yr of followup, respectively [5]. 

At present orthotopic liver transplantation is the only therapeutic option for patients with acute and chronic ESLDs. Liver transplantation, however, has the disadvantage of requiring lifelong immunosuppression and followup, with 10–15% of patients dying whilst on the waiting list due to the shortage of donated organs [6]. In 2005, only one-third of patients waiting for a liver transplant were transplanted [6]. With the number of donor organs likely to decrease over the coming decades, research into the alternative methods of treatment of whole-organ transplant is essential. Hepatocyte transplantation has been suggested as an alternative to liver transplantation, especially for hepatic disorders caused by inherited protein deficiency [7]. The widespread application of hepatocyte transplantation, however, is also limited by organ availability, by problems with viability of isolated hepatocytes after cryopreservation, and by the potential formation of hepatocyte aggregates during injection subsequently obstructing liver sinusoids and resulting in portal hypertension or fatal emboli.

Recent advances in the understanding of stem cell biology and plasticity have raised expectations for using stem cells as a new type of cellular therapy in regenerative medicine. In particular, adult hematopoietic stem-cell (HSC-) based treatment is evolving as a viable clinical alternative. These cells are capable of differentiating into many types of tissues, including skeletal and cardiac muscles, neuronal cells, pneumocytes, and hepatocytes [8]. Although stem cell therapy is not classically considered within the realm of clinical medicine, this technology will become increasingly important for clinicians in the future.

The BM compartment is largely made up of HSCs, committed progenitor cells, and noncirculating stromal cells called mesenchymal stem cells (MSC) which have the ability to develop into mesenchymal lineages [9, 10]. Haematopoietic stem cells are adult stem cells that can be identified by their ability to differentiate into all blood cell types and reconstitute the haematopoietic system in a host that has been lethally myeloablated [11]. It was previously thought that adult stem cells were lineage restricted, but recent studies have shown that BM-derived progenitors participate in the regeneration of ischaemic myocardium [12], damaged skeletal muscle [13], and neurogenesis [14], in addition to haematopoiesis. This paper focuses on the potential of HSCs in the management of CLD, concentrating on experimental models in animal and human tissue along with the current status of clinical trials. 

## 2. The Role of Bone Marrow Stem Cells (BMSCs) in Liver Repair

One of the first demonstrations of the ability of BMSCs to reconstitute liver was reported by Petersen and colleagues in 1999. Lethally irradiated female rats with induced hepatic injury, treated with 2-aminoacetylfluorine to prevent hepatic proliferation, and were rescued using bone marrow transplants from syngeneic males. The Y-chromosome markers dipeptidyl peptidase IV enzyme (DPPIV) and L21-6 antigen were used to identify liver cells of BM origin. This cross-sex model allowed the identification of male liver cells in the female rats' livers indicating that BM-derived HSCs have the capacity to transdifferentiate into hepatocytes [15].

Although evidence of transdifferentiation to hepatocytes is compelling from animal studies, few have examined this possibility in humans. Alison and associates detected Y-chromosome-positive cells in a retrospective analysis of the livers of 9 female recipients of bone marrow transplants from male donors. Cells were confirmed as being hepatocytes due to their expression of cytokeratin-8 [16]. The authors also looked for the presence of Y-chromosome-positive cells in 11 female livers transplanted to male recipients that were later removed due to recurrent disease, finding a number that expressed cytokeratin-8 (0.5%–2%). This confirmed that circulating extrahepatic stem cells colonise the liver [16]. 

## 3. Mechanism of Hepatocyte Regeneration

There is much controversy concerning the mechanism by which BMSCs contribute to hepatocyte regeneration or to liver repair. Transdifferentiation into hepatocytes represents genomic plasticity in response to the microenvironment and has been shown in several experiments *in vivo* [17–19]. However, some authors have proposed that conversion to hepatocytes may occur via cell fusion [20, 21]. The so-called “bystander effect” is postulated to be due to factors secreted by BMSCs that are chemoattracted to the site of injury, leading to the stimulation of mitosis of endogenous liver cells. This mechanism is thought to recruit endogenous BM for cardiac repair following myocardial infarction following administration of granulocyte colony-stimulating factor (G-CSF) [22]. 

 Other possible explanations for target organ regeneration and improvement in function include facilitating the release of vascular endothelial growth factor (VEGF) by stem cells, thus, increasing the blood supply to cells and helping to repair damaged tissue [23]. Stem cells may also act by up-regulating the Bcl-2 gene and suppressing apoptosis [24] or by suppressing inflammation in the diseased organ via the interleukin-6 (IL-6) pathway [25]. Both of these processes are thought to contribute to the regeneration of normal cells in the damaged organ. Finally, HSCs may stimulate tissue-specific stem cells, such as oval cells in the liver, facilitating regeneration of the target organ [26].

## 4. Animal Studies

Jang and colleagues transplanted enriched CD45^+^ HSCs into lethally irradiated mice treated with a single dose of carbon tetrachloride (CCl_4_) [19]. In this model, 7.6% of liver cells were of donor origin within 7 days of transplantation. There was early amelioration of liver disease with some improvement in liver function in transplanted mice compared to controls. The most promising study to date demonstrates liver disease reversal following transplantation of enriched HSCs into fumarylacetoacetate hydrolase- (FAH-) deficient mice, an animal model of tyrosinemia type I [27]. Bone marrow from metabolically competent donor mice was transplanted into a lethally irradiated FAH-deficient mouse strain, resulting in the proliferation of large numbers of donor LacZ^+^ hepatocytes and restoration of liver biochemical function. However, an animal study to investigate whether transplantation of HSCs CD34^+^ could improve hepatic fibrosis by their differentiation into hepatocytes found differing results [28]. HSCs from human umbilical cord blood were purified, transduced with a lentiviral vector containing the green fluorescent protein (GFP) gene, and injected via the portal vein into rats with liver cirrhosis induced by the four-month administration of thioacetamide. Rats were killed at 15 and 60 days following transplantation. Up to 37% and 22% fluorescent cells were observed in the blood of control and cirrhotic rats respectively, at 15 days after transplantation. At 60 days after transplantation; however, fluorescent cells were completely absent from the blood. Fluorescence was not detected in liver sections at either 15 or 60 days after transplantation. A polymerase chain reaction study to detect the GFP gene ruled out silencing of the transgene. These results suggest that the transplanted cells did not engraft in the liver and were eliminated from the rats. 

Oyagi and associates have transplanted MSCs, induced to adopt hepatocyte phenotype *in vitro*, intravenously into nonirradiated CCl_4_-damaged recipients and observed both a rise in serum albumin and a histological decrease in hepatic fibrosis [29]. Similarly, Jiang et al. transplanted ROSA26 mouse multipotent adult progenitor cells (MAPC) into nonobese diabetic/severe combined immunodeficiency disease (NOD/SCID) mice sacrificing them 4–24 weeks later. Recipient livers contained 5–10% donor cells colocalised with the hepatocyte markers CK18 and albumin. Since there was no noxious liver injury creating a donor cell survival advantage, there was no increase in the number of donor cells in the 6-month after transplant period [30].

Persistent injury has been found to induce efficient trans-differentiation of BMCs into functional hepatocytes [18]. *Green fluorescent protein-* (GFP-) transfected BMCs from nontreated mice injected into those with liver cirrhosis induced by CCl_4_ efficiently migrated into the periportal area of liver lobules after one day, repopulating 25% of the recipient liver by 4 weeks. In contrast, no GFP-positive BMCs were detected following transplantation into control mice with undamaged livers. Serum albumin levels were significantly elevated to compensate for chronic liver failure in BMC transplantation suggesting that recipient conditions and microenvironments are key factors for successful cell therapy using BMCs.

## 5. Clinical Studies

Several studies have demonstrated the presence of cells of bone marrow origin in the human liver. Alison and colleagues [16] elegantly demonstrated that adult human liver cells can be derived from stem cells originating in bone marrow. Analysing livers from female patients who had received a bone marrow transplantation from a male donor, they found Y-chromosome- and CK8- positive hepatocytes, thus, suggesting that extrahepatic stem cells can engraft in the liver. Theise et al. [31] also studied autopsy and liver biopsy tissue from recipients of sex-mismatched therapeutic bone marrow and orthotopic liver transplantations. They identified hepatocytes and cholangiocytes of bone marrow origin by immunocytochemistry staining for CK8, CK18, and CK19 and FISH analysis for the Y-chromosome. They found up to 43% of hepatocytes and 38% of cholangiocytes were engrafted, showing that these cells can be derived and differentiated from bone marrow to replenish the liver. Other studies, however, have found lower numbers. Korbling and associates [32], for example, confirmed bone marrow-derived hepatocytes in liver biopsies of sex-mismatched bone marrow transplantation, but these represented only 4%–7% of hepatocytes. Similarly, Ng et al. [33] showed that only a small proportion of hepatocytes (1.6%) were recipient derived in the liver allografts. The inconsistency of these studies may relate to the use of varying techniques and markers to identify recipient-derived hepatocytes in the transplanted patients.

Although studies have shown that bone marrow stem cells can give rise to hepatocytes, the use of bone marrow stem cells as therapeutic agents is still in its infancy. These studies generally involve the mobilisation of bone marrow stem cells using granulocyte colony-stimulating factor (G-CSF) or infusion of collected bone marrow stem cells, either peripherally or directly into the hepatic vasculature (Table 1). Our group conducted a phase I clinical trial of the infusion of CD34^+^ cells into the portal vein or the hepatic artery of five patients with chronic liver disease with no adverse effects [34]. Although these patients received relatively low numbers of cells (2 × 10^6^), a moderate improvement in serum bilirubin was seen in 3 of the 5 patients which lasted for more than 18 months [35]. Our experience is in keeping with the observations made by Am Esch II and associates, who in their first publication demonstrated increased liver regeneration in 3 patients following intra-portal administration of autologous CD133^+^ BM cells into the left lateral portal vein branches during right portal vein embolisation (PVE). By CT criteria, left lateral segment hypertrophy was 2.5-fold higher compared to 3 patients that had right PVE only [36]. In their second publication, which included patients from the first study, they recruited a total of 13 patients [37]. There was a significant increase in the daily liver growth in patients who had stem cell infusions in addition to PVE (*n* = 6) when compared to patients with PVE alone (*n* = 7). 

Terai and colleagues have also shown improvement in liver function following peripheral infusion of autologous BM cells in patients with liver cirrhosis. Nine patients who received a peripheral vein infusion of an average of 5.2 × 10^9^ autologous mononuclear cells (CD34^+^, CD45^+^, and ckit^+^) demonstrated significant improvement in the Child-Pugh Scores and serum levels of albumin. Liver biopsies were taken in 3 patients revealing an increase in proliferating cell nuclear antigen staining, an indirect marker of hepatocyte turnover [38]. Yannaki and associates have reported 2 patients with alcohol-induced liver cirrhosis treated with autologous mobilised HSCs [39]. Each patient underwent three rounds of G-CSF mobilisation and peripheral vein infusion of CD34^+^ cells. The procedure was well tolerated, and both patients improved their baseline Child-Turcotte-Pugh (CTP) and model for end-stage liver disease (MELD) scores during 30 months of followup. A further 2 patients with hepatitis-B-related decompensated liver cirrhosis treated with mobilised autologous peripheral blood monocytes (PBMC) also showed an improvement in serum albumin, bilirubin, alanine aminotransferase (ALT), aspartate aminotransferase (AST), and CTP scores for greater than one year following transfusion [40].

Lyra and associates performed a study on 10 patients with chronic end-stage liver disease, receiving committed progenitor cells and no BM cells via the hepatic artery. This study showed improvement in serum bilirubin, albumin, and international normalised ratio (INR) [41]. They went on to perform the first randomised controlled study of autologous BMC transplantation in liver disease [42]. Thirty patients were randomised to receive either a placebo or BMC, in the form of an autologous mononuclear cell preparation infused into the hepatic artery. After 3 months followup, the treated patients had a significant improvement in albumin compared to controls (16% versus 2%) and a significant reduction in their Child-Pugh status (−8% versus +4%). There was no change in the INR between the two groups. Gasbarrini et al. have also reported the successful use of autologous CD34^+^ BMSCs via the portal vein as a rescue treatment in a patient with drug-induced acute liver failure [43]. A liver biopsy performed at 20 days following infusion showed increased hepatocyte replication around necrotic foci; there was also improvement in synthetic liver function within the first 30 days. The patient, however, died secondary to multiorgan failure related to bacterial infection. In a study by Khan and colleagues, four patients with liver insufficiency were given G-CSF to mobilise stem cells. CD34^+^ cells (0.1 × 10^8^) were injected into the hepatic artery [44]. All patients showed improvements in serum albumin, bilirubin, and ALT after one month from the cell infusion.

In contrast to the previous studies, a trial of 4 patients with decompensated cirrhosis treated with CD34^+^ stem cells via the hepatic artery was stopped prematurely due to one patient developing nephropathy and hepatorenal syndrome secondary to radiocontrast [45]. The same group showed that MSC transplantation in a further 4 patients with decompensated liver cirrhosis, this time via a peripheral vein, was well tolerated and resulted in a MELD score improvement in two [46]. Another study injecting MSC into either peripheral veins or the portal vein of patients with ESLD having a MELD score ≥10 (*n* = 8) showed significant improvement in their MELD score [47]. Kim et al. reported significant improvement in serum albumin, quality of life, and the Child-Pugh Score in ten patients with advanced liver cirrhosis due to hepatitis B infection following autologous bone marrow infusion [48]. Finally, our group has published the results of administering autologous expanded mobilised adult BM CD34^+^ cells via the hepatic artery in 9 patients with alcoholic liver cirrhosis. Significant decreases in serum bilirubin, ALT, and AST levels were observed, whilst the Child-Pugh Scores and radiological ascites improved in 7 and 5 patients, respectively [49].

## 6. Limitations of Studies and Future Issues

Although all clinical trials to date have shown some improvement in liver function, it must be remembered that the natural history of cirrhosis tends to be variable. Thus, one would expect some patients to improve with time, particularly in compliant patients who can be followed up and remain abstinent from alcohol. The liver contains approximately 2.8 × 10^11^ hepatocytes, and the required mass of cells to correct a single enzyme biochemical defect is likely to be significantly less than that required for treatment of chronic or acute liver failure. There is evidence to suggest that transplantation of only 1–5% of the total liver mass may be sufficient to restore adequate functional activity [7, 50]. Cells can be delivered to patients via a peripheral vein, the portal vein, hepatic artery, or an intrasplenic injection. As both fulminant and chronic, liver failure requires the replacement of greater than 10% of functional liver; the cell mass required for transplantation will be significantly higher. The liver cell mass is restored primarily through division of mature hepatocytes. Stimulation of regeneration, such as a partial hepatectomy, promotes increases in carbamoyl phosphate synthetase I activity with subsequent liver hypertrophy [51]. This early experience suggests that this therapeutic approach has the potential of both enhancing and accelerating hepatic regeneration in a clinical setting. 

Unlike hepatocytes, the use of BMSCs for liver regeneration does not depend on the procurement of cadaveric livers whose donors are often immunologically disparate and also in short supply. The use of adult stem cells is attractive as it overcomes the moral and ethical barriers of ES cell manipulation. Further advantages of the use of BMSCs are that they are multipotent, there is already considerable experience in their use, they are easily accessible, and there is unlimited supply. Conversely, concerns have been raised about the adverse long-term effects of stem cell therapy. There is evidence to suggest that treatment with BMSCs may provide liver fibrogenic cells (hepatic stellate cells and myofibroblasts) which contribute to fibrosis and could have a deleterious effect on already decompensated cirrhotic livers [52, 53]. Similarly, there are concerns that hepatocellular carcinoma (HCC) originates from hepatic oval cells and BMSCs [54]. Much of the data concerning the malignant potential of BMSCs, however, originates from genetically modified rodent models and may not be present in humans [55]. 

New findings in adult stem cell biology are transforming our understanding of tissue repair raising hopes of successful regenerative hepatology. Although all clinical trials to date have shown some improvement in liver function and CD34^+^ cells have been used safely for BM transplantation for over 20 years, only randomised controlled clinical trials will be able to fully assess the potential clinical benefit of adult stem cell therapy for patients with liver insufficiency secondary to ALD.

## Figures and Tables

**Table 1:** Clinical trials of bone marrow stem cells in liver disease.

Reference	Patient group	Number (*n*)	Site injection	BM cells	Outcome
Am Esch II et al. [36]	Patients with liver cancer, no cirrhosis	Study: 3 Control: 3	Portal vein	Autologous, bone marrow, CD133^+^	2.5-fold increase in left lobe in study group on CT volumetry

Gordon et al. [34]	Hepatitis B or C Alcoholic cirrhosis and primary sclerosing cholangitis	5	Portal vein 3 Hepatic artery 2	Autologous peripheral blood, post-G-CSF CD34^+^	Improvement in albumin or bilirubin in 3/5 patients

Terai et al. [38]	Hepatitis B or C Cirrhosis	9	Peripheral vein	Autologous, iliac crest, unsorted BM	Improvement in the Child-Pugh Score and albumin

Yannaki et al. [39]	Alcoholic cirrhosis	2	Peripheral vein	Autologous Peripheral blood, post-G-CSF CD34^+^	Improvement in the Child-Pugh and MELD Score

Furst et al. [37]*	Patients with liver cancer, no cirrhosis	Study: 6 Control: 7	Portal vein	Autologous, bone marrow, CD133^+^	Increase in daily liver growth

Lyra et al. [41]	Alcoholic, hepatitis C, cholestatic and cryptogenic cirrhosis	10	Hepatic artery	Autologous, Iliac crest, unsorted BM	Improvement in Child's Pugh score, bilirubin, and albumin

Gasbarrini et al. [43]	Drug-induced acute liver failure	1	Portal vein	Autologous Peripheral blood, post-G-CSF CD34^+^	Improvement in clotting (PT), AST, and ALT up to 30 days Patient died of sepsis at day 60

Yan et al. [40]	Hepatitis B cirrhosis	2	Hepatic artery	Autologous peripheral blood monocytes post-G-CSF	Improvement in albumin, Bilirubin, AST, ALT, and the Child-Pugh Score

Mohamadnejad et al. [45]	Decompensated cirrhosis	4	Hepatic artery	Autologous bone marrow-haematopoietic CD34^+^ cells	Two patients showed minor improvement in albumin one had nephropathy and hepatorenal syndrome—trial terminated

Mohamadnejad et al. [46]	Decompensated cirrhosis	4	Peripheral vein	Autologous bone marrow mesenchymal stem cells	MELD Score improvement in 2 patients

Pai et al. [49]	Alcoholic liver cirrhosis	9	Hepatic artery	Autologous, post-G-CSF, CD34^+^ cells expanded *in vitro *	Significant improvement in bilirubin and liver enzymes

Khan et al. [44]	Hepatitis B or C	4	Hepatic artery	Autologous, post-G-CSF, CD34^+^ cells	Improvement in liver function

Kharaziha et al. [47]	Hepatitis B or C Alcoholic cirrhosis and Cryptogenic	8	Peripheral or portal vein	Autologous bone marrow mesenchymal stem cells	Significant improvement in MELD Score

Lyra et al. [42]	Hepatitis B or C Alcoholic and cryptogenic cirrhosis	Study: 15 Control: 15	Hepatic artery	Autologous mononuclear-enriched bone marrow cells	Improvement in liver function

Kim et al. [48]	Hepatitis B	10	Peripheral vein	Autologous bone marrow cells	Improvement in albumin, quality of life, and the Child-Pugh Score

*This includes Am Esch II et al. [36].
